# Active or Autoclaved *Akkermansia muciniphila* Relieves TNF-α-Induced Inflammation in Intestinal Epithelial Cells Through Distinct Pathways

**DOI:** 10.3389/fimmu.2021.788638

**Published:** 2021-12-16

**Authors:** Yuheng Luo, Cong Lan, Kunhong Xie, Hua Li, Estelle Devillard, Jun He, Li Liu, Jingyi Cai, Gang Tian, Aimin Wu, Zhihua Ren, Daiwen Chen, Bing Yu, Zhiqing Huang, Ping Zheng, Xiangbing Mao, Jie Yu, Junqiu Luo, Hui Yan, Quyuan Wang, Huifen Wang, Jiayong Tang

**Affiliations:** ^1^ Key Laboratory for Animal Disease-Resistance Nutrition of Ministry of Education of China, Animal Nutrition Institute, Sichuan Agricultural University, Chengdu, China; ^2^ Key Laboratory for Animal Disease-Resistance Nutrition and Feed of Ministry of Agriculture of China, Animal Nutrition Institute, Sichuan Agricultural University, Chengdu, China; ^3^ Key Laboratory of Animal Disease-Resistant Nutrition of Sichuan Province, Animal Nutrition Institute, Sichuan Agricultural University, Chengdu, China; ^4^ Center of Research for Nutrition and Health, Adisseo France SAS, Commentry, France; ^5^ College of Food Science and Technology, Nanjing Agricultural University, Nanjing, China; ^6^ College of Veterinary Medicine, Sichuan Province Key Laboratory of Animal Disease and Human Health, Key Laboratory of Environmental Hazard and Human Health of Sichuan Province, Sichuan Agricultural University, Chengdu, China

**Keywords:** *Akkermansia muciniphila*, inflammation, intestinal epithelial cells, TNF-α, apoptosis

## Abstract

Intestinal inflammation is a major threat to the health and growth of young animals such as piglets. As a next-generation probiotics, limited studies have shown that *Akkermansia muciniphila* could alleviate inflammation of intestinal epithelial cells (IECs). In this study, a TNF-α-induced inflammatory model of IPEC-J2 cells, the intestinal porcine enterocytes, was built to evaluate the effects of active or inactive *A. muciniphila* on the inflammation of IECs. The viability of IPEC-J2 cells was the highest when treated with active (10^8^ copies/mL) or inactive (10^9^ copies/mL) *A. muciniphila* for 7.5 h (*P* < 0.01). Treated with 20 ng/mL of TNF-α and followed by a treatment of *A. muciniphila*, the mRNA level of proinflammatory cytokines (*IL-8, IL-1β*, *IL-6* and *TNF-α*) was remarkably reduced (*P* < 0.05) along with the increased mRNA level of tight junction proteins (*ZO-1* and *Occludin*, *P* < 0.05). Flow cytometry analysis showed that active or inactive *A. muciniphila* significantly suppressed the rate of the early and total apoptotic of the inflammatory IPEC-J2 cells (*P* < 0.05). According to results of transcriptome sequencing, active and inactive *A. muciniphila* may decline cell apoptosis by down-regulating the expression of key genes in calcium signaling pathway, or up-regulating the expression of key genes in cell cycle signaling pathway. And the bacterium may alleviate the inflammation of IECs by down-regulating the expression of PI3K upstream receptor genes. Our results indicate that *A. muciniphila* may be a promising NGP targeting intestinal inflammation.

## Introduction

The gastrointestinal epithelium represents the direct contact surface between the body and external environment. Its selective permeability allows the appropriate absorption of nutrients and water, while prevents the invasion of noxious molecules and microorganisms in the lumen (1). The cellular architecture of the intestines features a continuous monolayer of intestinal epithelial cells (IECs) that represent the first defensive barrier against environmental and microbial attacks by carrying out several critical innate immune functions, and many factors such as pathogens can cause the inflammation or apoptosis of IECs (2). Normal intestinal functions are closely linked to the balance of proliferation and apoptosis of IECs. An obvious example for the dysfunction of IECs is the intestinal inflammation caused by social, environmental or dietary stress in young animals, which leads to a growth defect (3). Antibiotics, used as growth promoters, are also recognized as effective anti-inflammatory additives for animals (4). However, the increasing number of antibiotic-resistant bacterial strains in animal gut accelerates research to develop alternative methods for the prevention and therapy of intestinal inflammation in young animals. Amongst those alternatives, probiotics, could modulate inflammation, by regulating for instance the production of cytokines and IgA (5, 6).

Compared with the first-generation probiotics (FGPs), the next-generation probiotics (NGPs) which are anaerobic gut commensal microbes that have been shown to suppress mucosal inflammation (7, 8). Of all NGPs, *Akkermansia muciniphila*, a newly isolated Gram-negative bacterium (9), has been shown to have beneficial effect on type II diabetes, obesity and colorectal cancer (10–14). It is also reported that *A. muciniphila* enhances the thickness of the mucus layer in obese mice induced by high-fat diet and the integrity of IECs in several *in vitro* studies (10, 15), and may contribute to the epithelial homeostasis and cell fate (16). Today, these beneficial effects were mainly observed using active *A. muciniphila*. Some special structures of *A. muciniphila*, such as a specific protein Amuc_1100 isolated from the outer membrane or *A. muciniphila*-derived extracellular vesicles (AmEVs), can also modulate the intestinal barrier and immune responses of host (17, 18). These limited results indicate that *A. muciniphila* compounds may alleviate the inflammation, suggesting that having active cells might not be necessary to obtain an effect on inflammation.

The aim of the current study was to assess the effects of active or inactive *A. muciniphila* in an *in vitro* inflammatory model based on IPEC-J2 cells which is closed to human physiology and has been widely used as an *in vitro* model in physiological, pathological and nutritional studies (19). A specific focus was put on monitoring the expression of inflammatory cytokines and apoptosis, as well as the transcriptome of the cells. These data were then discussed to provide new thoughts about the application of *A. muciniphila* as a NGP in animal feed or human food.

## Materials and Methods

### Bacterium Preparation and Growth Curve

*A. muciniphila* (strain DSM 22959) was cultured in brain heart infusion broth (BHI) (Qingdao Rishui Bio·Technology Co., Ltd, Qingdao, China) (9). The medium was prepared with double distilled water (ddH_2_O) under a complete anaerobic condition with high-purity carbon dioxide. Then each of 10 mL medium was allocated in a 20 mL tubes and immediately sealed with butyl rubber stopper and aluminum crimp cap followed by an autoclave at 121°C for 20 min. All tubes inoculated with *A. muciniphila* were incubated at 37°C. To establish the growth curve of *A. muciniphila*, each of 500 µL culture was firstly collected at 6, 12, 18, 24, 30, 36, 42 and 48 h and then centrifuged at 10,000 × *g* for 15 min, respectively. Bacterial growth was measured by qPCR. Genomic DNA was extracted using a described method (20). Specific primers, AM1 (5’-CAGCACGTGAAGGTGGGGAC-3’) and AM2 (5’-CCTTGCGGTTGGCTTCAGAT-3’) (21), were used to amplify partial 16s rRNA gene of *A. muciniphila*. The real-time PCR was performed on a CFX96TM Real-Time System (Bio-Rad, USA) in triplicate, and the 10 µL reaction solution included 5 µL SYBR Premix EX Taq (TaKaRa, Dalian, China), 1 µL each of the primers (10 µM), 1 µL DNA template and 3 µL ddH_2_O. The PCR reaction was performed as follows: a pre-denaturation at 95°C for 10min, a total of 40 cycles of 95°C for 15 s, 60°C for 40 s and 72°C for 30 s, as well as a final extension at 72°C for 5 min.

### The Preparation of Active and Inactive *A. muciniphila*

**Figure 1:**
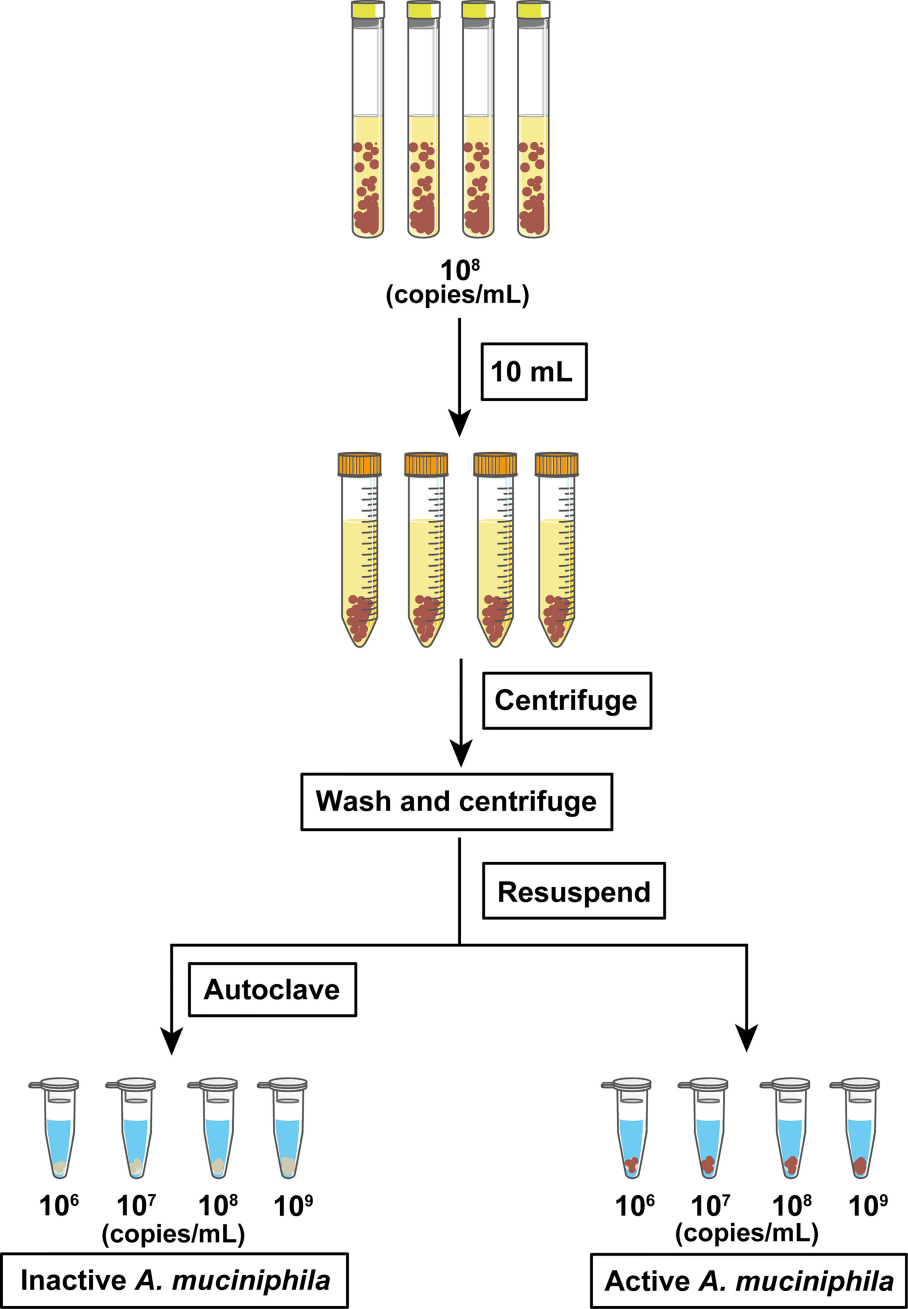
Flow chart of the preparation of active and autoclaved inactive *A. muciniphila*.

The process for the preparation of active and inactive *A. muciniphila* cells is shown in **Figure 1**. In detail, 10^8^ copies/mL was collected and centrifuged at 5,000 × *g* for 5 min. The pellet containing the cells of *A. muciniphila* was washed with 1 × anaerobic phosphate-buffered saline (PBS) (Solarbio, Beijing, China) and centrifuged for three times and then resuspended into 1 mL cell culture medium (descripted below) to obtain the concentrated active *A. muciniphila* (different concentrations from 10^6^ to 10^9^ copies/mL). Aliquots of those diluted cells were then autoclaved at 120°C for 30 min to obtain inactive *A. muciniphila* cells. For the following survival test, active or inactive *A. muciniphila* was inoculated and cultured in BHI medium, respectively. Then, the OD_600_ value of culture containing the two types of bacteria were tested at 3 h, 6 h, 18 h. To investigate whether autoclave caused damage to the integrity of the bacterium, the prepared cells (active and inactive) of *A. muciniphila* were collected and fixed for 2 h in a solution with 4% glutaraldehyde away from light. The specimens were dehydrated in absolute alcohol and critical-point dried with carbon dioxide. Finally, each sample were glued onto a sample holder using a carbon adhesive tab and sputter-coated with 10 nm platinum, and observed with a field emission scanning electron microscope (JEOL 7500 F) at 15 kV.

### Cell Culture and TNF-α-Induced Inflammation Model

To investigate the role of *A. muciniphila* on inflammatory IECs, we used tumor necrosis factor-α (TNF-α) as an inducer of inflammatory response on IPEC-J2 cells, the intestinal porcine enterocytes isolated from the jejunum of a neonatal, as described previously (22).

IPEC-J2 cells used in the current study were donated by Dr. Per Torp Sangild from University of Copenhagen (Denmark) and cultured in a complete medium containing Dulbecco’s Modified Eagle Medium: F-12 (DMEM-F12) (HyClone, USA), 10% (v/v) fetal bovine serum (FBS) (HyClone, USA) which is more suitable in this experiment (19, 23, 24) and 1% (v/v) Penicillin-Streptomycin (Solarbio, Beijing, China). 1×10^5^ cells/well were seeded and grown at 37°C in a CO_2_ incubator (5% v/v). When the cell fusion rate reaches 80%, the IPEC-J2 cells were treated with DMEM-F12 and starved for 12 h. To construct an inflammatory response model, the prepared IPEC-J2 cells were treated with 0, 10, 20, 40, 80 and 120 ng/mL of Recombinant Susscrofa TNF-α (Raybiotech, Inc Cat.# 230-00875) for 48 hours, respectively. Next, the mRNA level of Interleukin-1β (IL-1β), Interleukin-6 (IL-6) and TNF-α was determined using real-time PCR to assess whether the inflammatory model was built successfully, combining observed morphology of the cells with an inverted microscope (NiKon Ts100, C-W 10× (F.O.V. 22mm), Japan).

### Determination of Optimal Concentrations of *A. muciniphila* and Experimental Design

To determine the cell viability of IPEC-J2 cells, an Enhanced Cell Counting Kit-8 (CCK-8) (Biyuntian, Shanghai, China) was used. In brief, the activated IPEC-J2 cells were seeded in a 96-well plate (Corning, New York City, USA) at a concentration of 1×10^5^ cells per well for 48 h. The prepared living (active) and inactive *A. muciniphila* at 10^6^, 10^7^, 10^8^ and 10^9^ copies/mL were then inoculated into the cells in triplicate and incubated at 37°C for 2.5, 5, 7.5 and 10 h, respectively. At the end of the incubation, 10 µL CCK-8 solution was added to each well and continuously incubated for 2 h. The absorbance for each well was then measured at a wavelength of 450 nm using the Absorbance Microplate Reader (Molecular Devices, SpectraMax 190, USA). The optimum concentration of *A. muciniphila* to apply on IPEC was then confirmed *via* the results of cell viability of IPEC-J2 cells (results are shown below).

To investigate the protection of active and inactive *A. muciniphila* on IPEC-J2 cells, a total of 6 treatments were included. IPEC-J2 Cells in the negative control (CON) were treated with PBS, while cells in the positive control (TNF) were challenged with Recombinant Susscrofa TNF-α. The remaining 4 treatments included the following: IPEC-J2 cells with the optimum concentration (determined as described above) of active *A. muciniphila* (A), IPEC-J2 cells with the optimum concentration (determined as described above) of inactive *A. muciniphila* (IA), IPEC-J2 cells challenged with TNF-α and then incubated with active *A. muciniphila* (TNF+A), IPEC-J2 cells challenged with TNF-α and then incubated with inactive *A. muciniphila* (TNF+IA). Of these treatments, A and IA were designed to investigate the effect of both active and inactive *A. muciniphila* on normal IECs, TNF+A and TNF+IA aimed to assess the possible remission of inflammatory IECs by *A. muciniphila*.

### Real-Time PCR to Quantify Inflammatory Response and Barrier Function Related Genes in IPEC-J2 Cells

In this study, the relative expression of four genes encoding inflammation related cytokines and two tight junction proteins were selected as the markers of inflammation. The specific primers for *IL-1β*, *IL-6* and *TNF-α* were designed with Primer Primer5 software, while the primers for *IL-8* (22), zonula occludens-1 (*ZO-1*) (25), *Occludin* (26) and the three house-keeping genes, *β-actin* (27), *GAPDH* (28) and *TBP* (29) were referred to existing researches (**Table 1**). The total RNA of the collected IPEC-J2 cells was extracted using a TRIzol reagent (Invitrogen, Carlsbad, California, USA). Then, each RNA sample was reverse-transcribed into cDNA using PrimeScript RT reagent kit (TaKaRa, Dalian, China) after detection of RNA purity and integrity by a NanoDrop 2000 spectrophotometer (Thermo Fisher Scientific, Massachusetts, USA) and 1.5% agarose gel electrophoresis. Real-time PCR was also performed on a CFX96TM Real-Time System (Bio-Rad, USA) in triplicate. The 25 μL reaction solution consisted of 12.5 μL SYBR Premix EX Taq (TaKaRa, Dalian, China), 0.5 μL of each primer (10 μM), 2 μL cDNA template and 9.5 μL ddH_2_O. Procedures of the PCR reaction included a pre-denaturation at 95°C for 30 s followed by 40 cycles of 95°C for 5 s and 60°C for 34 s. The data of targeting genes were then normalized with house-keeping genes, and the relative expression of each gene was calculated using the 2^-△△Ct^ method (30).

**Table 1:** Primers for genes quantified with real-time PCR in the current study.

Gene*^a^*	Accession No.	Primer sequences*^b^* (5’ - 3’)	product size (bp)	Reference
β-actin	XM_003124280.5	F:TGGAACGGTGAAGGTGACAGC	177	(27)
		R:GCTTTTGGGAAGGCAGGGACT		
GAPDH	NM_001206359.1	F: TCGGAGTGAACGGATTTGGC	147	(28)
		R: TGCCGTGGGTGGAATCATAC		
TBP	DQ178129	F: GATGGACGTTCGGTTTAGG R: AGCAGCACAGTACGAGCAA	124	(29)
IL-8	X61151.1	F: AGTGGACCCCACTGTGAAAA	102	(22)
		R: TACAACCTTCTTCTGCACCCA		
IL-1β	NM_214055.1	F: GTGATGCCAACGTGCAGTCT	97	this study
		R:AGGTGGAGAGCCTTCAGCAT		
IL-6	NM_214399.1	F:AGGGAAATGTCGAGGCTGTGC	112	this study
		R: CCGGCATTTGTGGTGGGGTT		
TNF-α	NM_214022.1	F: TTCGAGGTTATCGGCCCCCA	114	this study
		R: GTGGGCGACGGGCTTATCTG		
ZO-1	XM_005659811.1	F: CAGCCCCCGTACATGGAGA	114	(25)
		R:GCGCAGACGGTGTTCATAGTT		
Occludin	NM_001163647.2	F:CTACTCGTCCAACGGGAAAG	158	(26)
		R: ACGCCTCCAAGTTACCACTG		

a GAPDH, glyceraldehyde 3-phosphate dehydrogenase; TBP, TATA box binding protein.

b F, forward; R, reverse.

### Detection of Apoptosis of the Cells

The apoptosis of IPEC-J2 cells was detected using an Annexin V-Fluorescein isothiocyanate isomer/Propidium Iodide (Annexin V-FITC/PI) kit (Beyotime, Shanghai, China) according to the manufacturer’s instructions. Shortly, the medium and cells in each well were collected at the end of the incubation, and then treated with 2 mL 0.25% ethylenediaminetetraacetic acid (EDTA)-free pancreatin (Beyotime, China) for 2 min. The solution was then collected into a 10 mL centrifugal tube and centrifuged at 1,000 × *g* for 5 min. Cells were then resuspended in 100 μL 1 × Binding Buffer to reach a final concentration of 1 × 10^5^ cells/mL. Next, 5 μL FITC Annexin V and 5 μL PI were added into the tube respectively, and the mixture was incubated at room temperature for 15 min away from light. Finally, 400 μL 1 × Binding Buffer was added to each tube and cell apoptosis was analyzed using a flow cytometry (FACSVerse, BD, USANIK) within 1 h.

### Construction of RNA Library and Bioinformatics Analysis

Total RNA of each sample was extracted using TRIzol (Invitrogen, USA), and the quality of extracted RNA was assessed using the RNA Nano 6000 Assay Kit of the Bioanalyzer 2100 system (Agilent Technologies, CA, USA). Approximate 1μg RNA of each sample was used for the preparation of library. The clustering of the index-coded samples was performed on a cBot Cluster Generation System using TruSeq PE Cluster Kit v3-cBot-HS (Illumia). Then, the prepared RNA samples were sequenced on an Illumina Novaseq platform and 150 bp paired-end reads were generated. All the downstream analyses were based on the clean data with high quality. FeatureCounts v1.5.0-p3 was used to count the reads numbers mapped to each gene. The FPKM (Fragments per Kilobase Million) of each gene was calculated based on the length of the gene and reads count mapped to this gene. Principal component analysis (PCA) was performed and visualized using the DESeq (2012) R package.

Differential expression genes (DEGs) were filtered according to the following parameters: *P* value < 0.05, and log2 fold change > 1 or < –1 in each pairwise comparison. The differential expression genes in Kyoto Encyclopedia of Genes and Genomes (KEGG) pathways were annotated using KOBAS 3.0 (http://kobas.cbi.pku.edu.cn/kobas3/genelist/) and visualized using the ggplot2 R package. The DEGs were annotated using STRING (https://cn.stringdb.org) and the protein interaction network was visualized using Cytoscape (3.7.1). All sequences were uploaded to NCBI database with an accession number (PRJNA752872).

### Statistical Analysis

Data were analyzed using a one-way ANOVA with Holm-Sidak’s multiple comparison (e.g., the optimum concentration of Recombinant Susscrofa TNF-α) or Tukey’s multiple comparison (e.g., the mRNA level of genes and the rate of apoptosis). The multiple t-test was used when comparing only two groups (e.g., the bacteria viability test and the processing time of TNF-α on the IPEC-J2 cells). The viability of IPEC-J2 cells to active or inactive *A. muciniphila* was analyzed using a two-way ANOVA with Sidak’s multiple comparison. Data were shown as mean ± standard error of the mean (SEM). Differences were considered to be significant when *P* < 0.05. Statistical analysis was performed using a GraphPad Prism 8 software (GraphPad Software, La Jolla, USA).

## Results

### The Inflammatory Response of IPEC-J2 Cells Induced by Recombinant Susscrofa TNF-α

**Figure 2:**
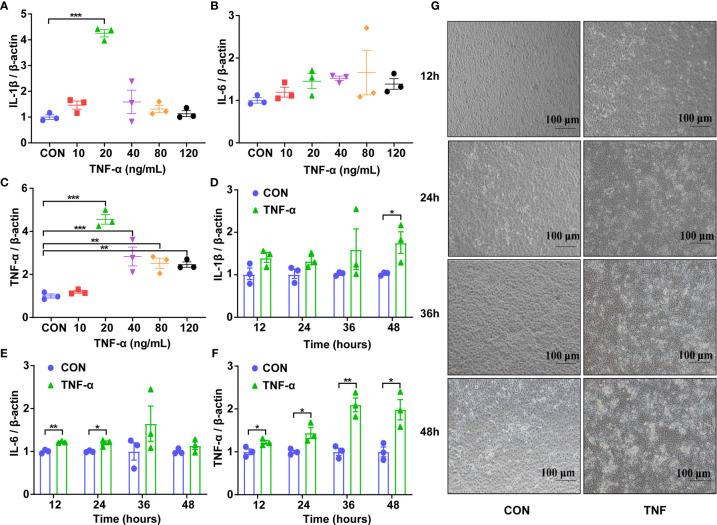
Expression of genes encoding inflammatory cytokines and the representative morphology of IPEC-J2 cells in different groups. **(A)** The mRNA level of *IL-1β* of cells challenged with Recombinant Susscrofa TNF-α with different concentrations (0, 10, 20, 40, 80, 120 ng/mL) for 48 h. **(B)** The mRNA level of *IL-6* of cells challenged with TNF-α with different concentrations (0, 10, 20, 40, 80, 120 ng/mL) for 48 h. **(C)** The mRNA level of *TNF-α* of cells challenged with TNF-α with different concentrations (0, 10, 20, 40, 80, 120 ng/mL) for 48 h. **(D)** The mRNA level of *IL-1β* of cells challenged with 20 ng/mL TNF-α for 12, 24, 36 and 48 h. **(E)** The mRNA level of *IL-6* of cells challenged with 20 ng/mL TNF-α for 12, 24, 36 and 48 h. **(F)** The mRNA level of *TNF-α* of cells challenged with 20-ng/mL TNF-α for 12, 24, 36 and 48 h. Data are represented as mean ± SEM (n = 3). ^*^ *P* < 0.05, ^**^ *P* < 0.01, ^***^ *P* < 0.001. CON, cells without TNF-α treatment. Results represented one of the three independent experiments. **(G)** The representative images of IPEC-J2 cells challenged with 20 ng/mL Recombinant Susscrofa TNF-α photographed by an inverted microscope under bright field at 12, 24, 36 and 48 h, respectively. The scale bar is 100 μm.

First, the appropriate concentration of Recombinant Susscrofa TNF-α to be applied on IPEC-J2 was determined. Cells were treated with TNF-α at 10, 20, 40, 80 and 120 ng/mL after 48 h, the expression of *IL-1β, IL-6* and *TNF-α* were measured. The highest response of *IL-1β* and *TNF-α* was obtained with the concentration of 20 ng/mL of TNF-α (*P* < 0.001, **Figures 2A, C**). *IL-6* was only increased with the concentration of 80 ng/mL TNF-α (**Figure 2B**). Taking these data together, the dose of 20 ng/mL TNF-α was chosen for further studies. To confirm the optimal processing time, the IPEC-J2 cells were then treated with 20 ng/mL TNF-α for 12, 24, 36, and 48 h. In the TNF-α-treated cells, the expression of the cytokines was increased when compared to the control at 48h for *IL-1β* (*P* < 0.05, **Figure 2D**), 12 and 24 h for *IL-6* (*P* < 0.01 or 0.05, **Figure 2E**), as well as 12, 24, 36 and 48h for *TNF-α* (*P* < 0.01 or 0.05, **Figure 2F**). Meanwhile, the bright-field images (**Figure 2G**) of inverted microscope showed that the integrity of those TNF-α-challenged cells was destroyed with obviously increased gap and the volume of nucleus compared to the control. Particularly, the IPEC-J2 cells treated with 20 ng/mL for 48 h showed the highest degree of cell destruction and the most cellular fragments, which are typical characteristics of apoptosis. Taking these data together, the incubation of 48 h with 20 ng/mL TNF-α was chosen to test the effects of *A. muciniphila* on stressed IPEC-J2 cells.

### The Cell Viability of IPEC-J2 Cells With Active or Inactive *A. muciniphila*

**Figure 3:**
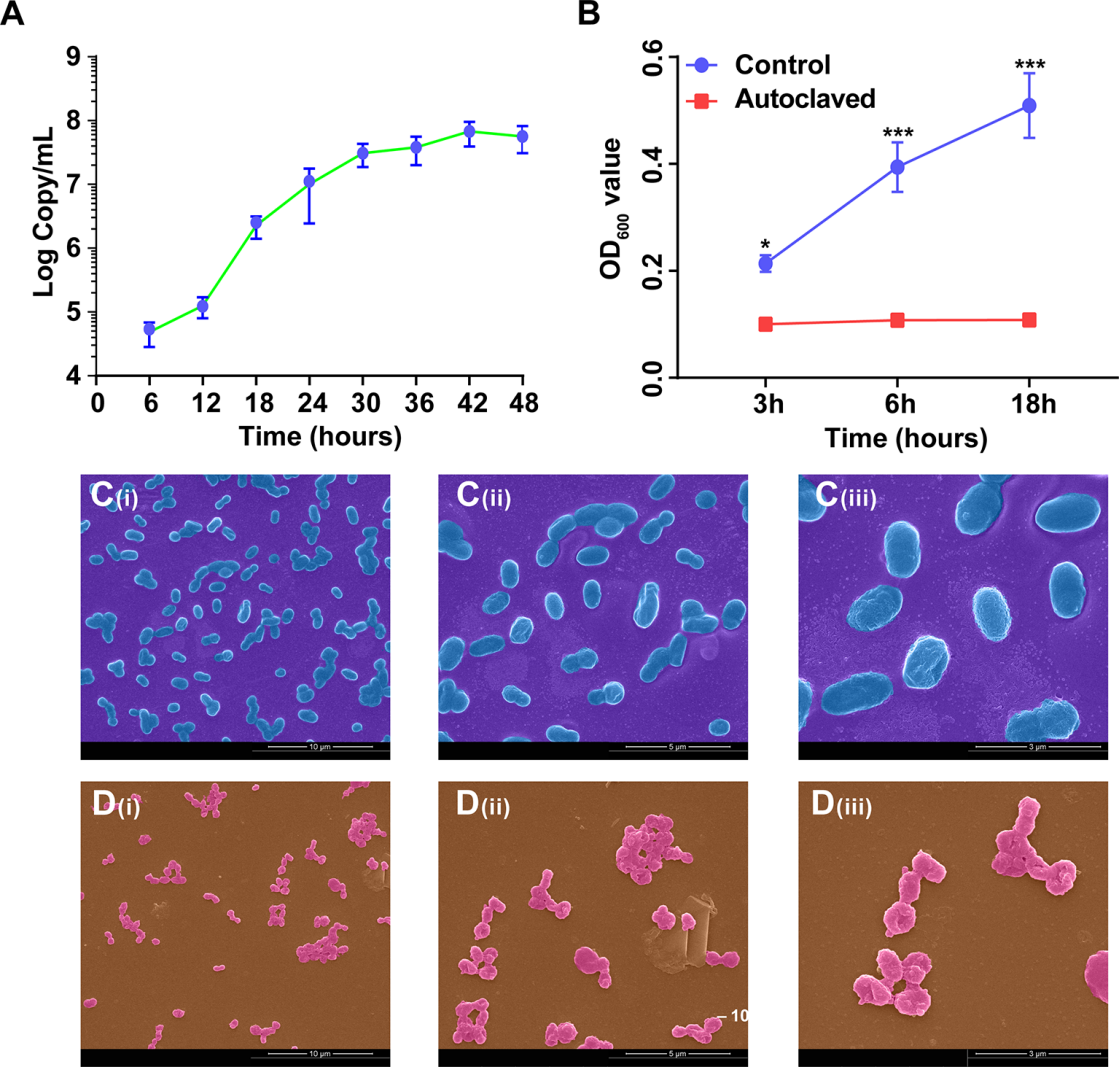
The growth curve and cellular morphology of *A. muciniphila*. **(A)** The growth curve of *A. muciniphila* cultured in BHI medium. **(B)** The growth curve of *A. muciniphila* with (Autoclaved) or without (Control) autoclaving in BHI medium within 18 hours. OD_600_ value. **(C)** SEM images of active *A. muciniphila*. **(i)** Bar, 10 µm. magnification times, × 5,000. **(ii)** Bar, 5 µm. magnification times, × 10,000. **(iii)** Bar, 3 µm. magnification times, × 20,000. **(D)** SEM images of inactive *A. muciniphila*. **(i)** Bar, 10 µm. magnification times, × 5,000. **(ii)** Bar, 5 µm. magnification times, × 10,000. **(iii)** Bar, 3 µm. magnification times, × 20,000. Data are expressed as mean ± SEM (n = 3). ^*^ *P* < 0.05, ^***^ *P* < 0.001. Results represented one of the two independent experiments.

According to the growth curve based on copy numbers (**Figure 3A**), the growth of *A. muciniphila* in BHI medium reached a plateau at 48 h. To verify whether *A. muciniphila* was effectively inactivated by the autoclave treatment, we detected the OD value of the bacterium (pure cells without medium) during 18 hours. The non-autoclaved *A. muciniphila* showed a growth at 6 and 18 h (*P* < 0.001, **Figure 3B**), whereas the autoclaved cells did not grow during the same period (*P* > 0.05, **Figure 3B**).

According to the profiles of scanning electron microscope (**Figures 3C, D**), the cellular morphology of active and inactive *A. muciniphila* showed less different. Specifically, the cells of active *A. muciniphila* were oval-shaped, owned complete and smooth cell walls, and the typical cell of autoclaved *A. muciniphila* showed irregular shape, sticked together, partially collapsed, accidented and even broken.

**Figure 4:**
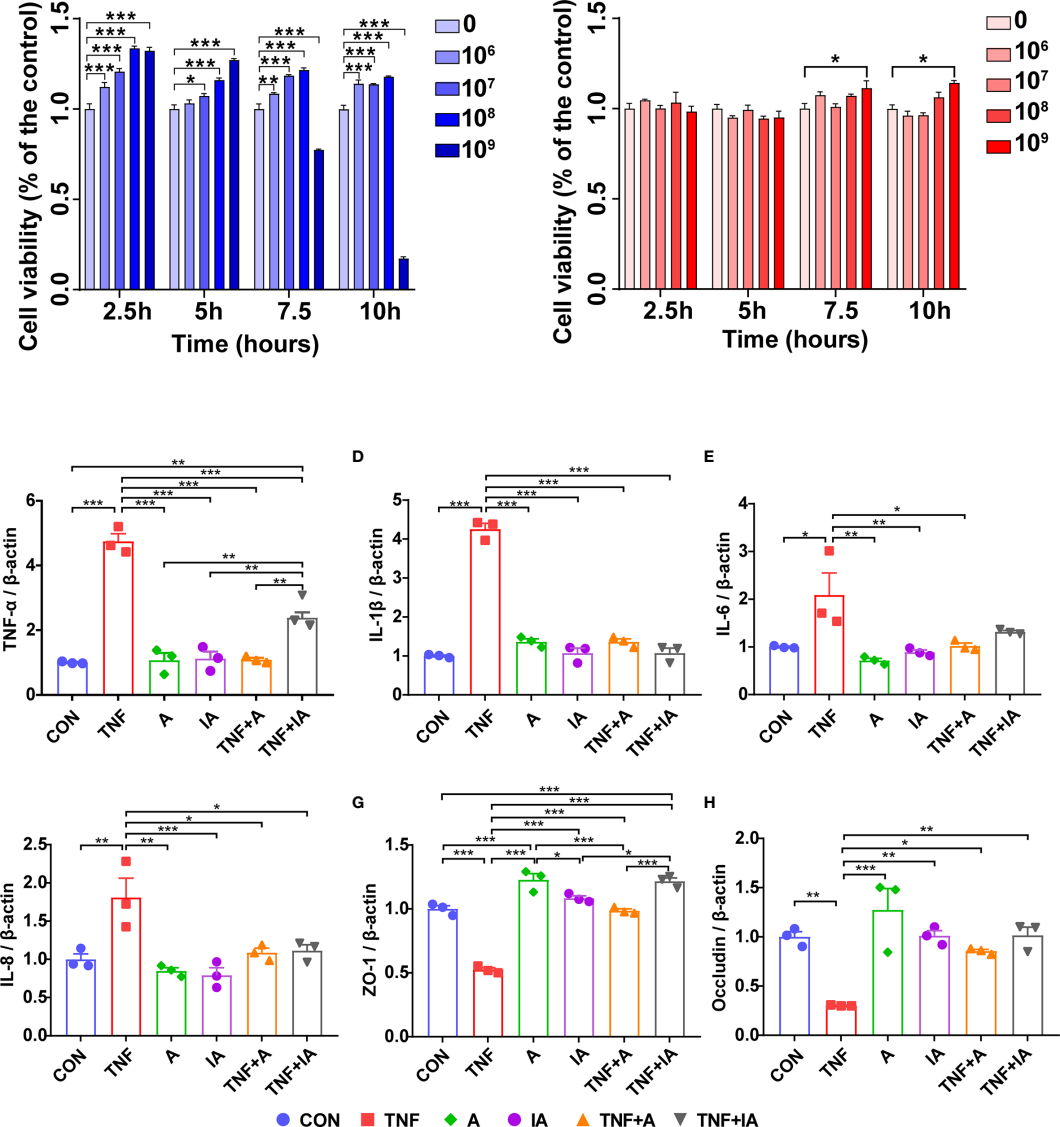
The cell viability of IPEC-J2 cells treated with active or inactive *A. muciniphila*. and mRNA level of inflammation related cytokines and tight junctions of the IPEC-J2 cells in different groups. **(A)** The viability of IPEC-J2 cells co-cultured with living (active) *A. muciniphila* at 0, 10^6^, 10^7^, 10^8^ and 10^9^ copies/mL for 2.5, 5, 7.5 and 10 h, respectively. **(B)** The viability of IPEC-J2cells co-cultured with inactive (autoclaved) *A. muciniphila* at 0, 10^6^, 10^7^, 10^8^ and 10^9^ copies/mL for 2.5, 5, 7.5 and 10 h, respectively. **(C)** The mRNA level of TNF-α of IPEC-J2 cells in different groups. **(D)** The mRNA level of IL-1β of IPEC-J2 cells in different groups. **(E)** The mRNA level of IL-6 of cells in different groups. **(F)** The mRNA level of IL-8 of IPEC-J2 cells in different groups. **(G)** The mRNA level of Occludin of IPEC-J2 cells in different groups. **(H)** The mRNA level of Occludin of IPEC-J2 cells in different groups. CON, cells treated with PBS for 48 h. TNF, cells challenged with 20 ng/mL Recombinant Susscrofa TNF-α for 48 hours. A, cells co-cultured with 10^8^ copies/mL active * A. muciniphila* for 48 h. IA, cells co-cultured with10^9^ copies/mL inactive *A. muciniphila* for 48 h. TNF+A, cells challenged with 20 ng/mL TNF-α for 40.5 h and then co-cultured with 10^8^ copies/mL active *A. muciniphila* for 7.5 h. TNF+IA, cells challenged with 20 ng/mL TNF-α for 40.5 h and then co-cultured with 10^9^ copies/mL inactive *A. muciniphila* for 7.5 h. ^*^ *P* < 0.05, ^**^ *P* < 0.01, ^***^ *P* < 0.001. Data are expressed as mean ± SEM (n = 3). Results represented one of the three independent experiments.

To determine the viability of IPEC-J2 cells in presence of *A. muciniphila*, the IPEC-J2 cells were co-cultured with active or autoclaved *A. muciniphila* applied at 10^6^ to 10^9^ copies/mL for 2.5, 5, 7.5 and 10 hours. Compared to non-treated IPEC-J2 cells, the viability of IPEC-J2 cells co-cultured with active *A. muciniphila* was increased for all times and all concentrations of the bacteria (*P* < 0.05 or 0.01 or 0.001, **Figure 4A**), except for the concentration of 10^9^ of active *A. muciniphila* for 7.5 and 10 h which led to a significant decrease in viability of IPEC-J2 cells (*P* < 0.001, **Figure 4A**). When treated with inactive *A. muciniphila*, IPEC-J2 showed an increased viability for a concentration of 10^9^ of inactive *A. muciniphila* for 7.5 and 10 h (*P* < 0.05, **Figure 4B**). From these results, the optimum concentration of active and inactive *A. muciniphila* should be 10^8^ and 10^9^ copies/mL respectively, and the incubation time with IPEC-J2 cells should be 7.5 h.

### The Gene Expression of Cytokines and Tight Junction Proteins (TJs) in IPEC-J2 Cells

In order to assess the effects of *A. muciniphila* on IECs, the normal or TNF-α challenged IPEC-J2 cells were co-cultured with active or inactive *A. muciniphila*. According to the results of real-time PCR, the expression of *TNF-α* (*P* < 0.001, **Figure 4C**), *IL-1β* (*P* < 0.001, **Figure 4D**), *IL-6* (*P* < 0.05, **Figure 4E**) and *IL-8* (*P* < 0.001, **Figure 4F**) showed increased in the cells in group TNF, while the expression of *ZO-1* (**Figure 4G**) and *Occludin* (**Figure 4H**) in these cells was decreased compared to CON group (*P* < 0.01 or 0.001). Comparing with cells in group TNF, the mRNA levels of *TNF-α* (**Figure 4C**), *IL-1β* (**Figure 4D**) and *IL-8* (**Figure 4F**) of the cells in groups A, IA, TNF+A and TNF+IA, as well as the mRNA level of *IL-6* (**Figure 4E**) of cells in groups A, IA and TNF+A were reduced (*P* < 0.05 or 0.01 or 0.001). The expression of *ZO-1* (**Figure 4G**) and *Occludin* (**Figure 4H**) of the cells co-cultured with both active and inactive *A. muciniphila* was higher than cells in TNF group (*P* < 0.05 or 0.01 or 0.001).

### The Apoptosis Rate of the IPEC-J2 Cells With Different Treatment

**Figure 5:**
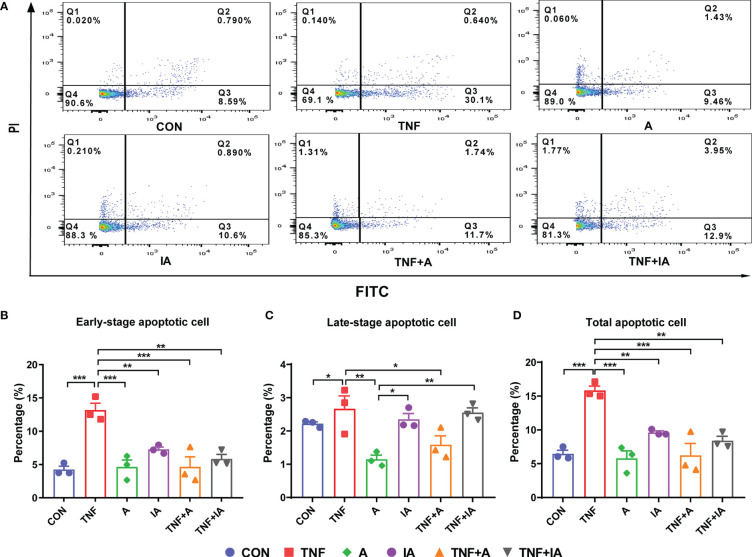
The apoptosis of IPEC-J2cells in different groups. **(A)** The profile of flow cytometry with an annexin V-FITC/PI kit of the IPEC-J2 cells in different groups. Each of the frames is divided into four quadrants: Q1, necrotic cells. Q2, cells in the late-stage of apoptosis. Q3, cells in the early-stage of apoptosis. Q4, normal cells. **(B)** The rate of IPEC-J2 cells in the early stage of apoptosis in different groups. **(C)** The rate of IPEC-J2 cells in the late stage of apoptosis in different groups. **(D)** The rate of total apoptosis of IPEC-J2 cells in different groups. Data are represented as mean± SEM (n = 3). ^*^ *P* < 0.05, ^**^ *P* < 0.01, ^***^ *P* < 0.001. CON, cells treated with PBS for 48 h. TNF, cells challenged with 20 ng/mL Recombinant Susscrofa TNF-α for 48 hours. A, cells co-cultured with 10^8^ copies/mL active *A. muciniphila* for 48 h. IA, cells co-cultured with 10^9^ copies/mL inactive *A. muciniphila* for 48 h. TNF+A, cells challenged with 20 ng/mL TNF-α for 40.5 h and then co-cultured with 10^8^ copies/mL active *A. muciniphila* for 7.5 h. TNF+IA, cells challenged with 20 ng/mL TNF-α for 40.5 h and then co-cultured with 10^9^ copies/mL inactive *A. muciniphila* for 7.5 h.

The degree of cellular damage can be reflected by the rate of apoptosis. In the current study, an annexin V-FITC/PI assay with flow cytometry was performed to investigate whether *A. muciniphila* could attenuate the apoptosis induced by TNF-α in IPEC-J2 cells. Results showed that compared with CON cells, the early-stage apoptosis (**Figures 5A, B**) of the cells in groups TNF (*P* < 0.001) was increased (**Figures 5A, B**). While the early-stage apoptosis of the cells in groups A, IA, TNF+A and TNF+IA was decreased compared to those cells in TNF group (*P* < 0.01 or 0.001).

Comparing with cells in CON group, the treatment of TNF-α increased the total apoptosis rate of the IPEC-J2 cells (*P* < 0.001, **Figure 5**). However, the treatment of active (TNF+A) *A. muciniphila* and group A and IA was found to reduce the total apoptosis rate of the TNF-challenged cells compared to TNF group (*P* < 0.01 or 0.001, **Figure 5D**). Meanwhile, the late-stage apoptosis of cells in group IA and TNF+IA was higher than those in other group (*P* < 0.05 or 0.01, **Figures 5A, C**).

### Expression of Genes in the Cells With Different Treatment Revealed by Transcriptome Analysis

**Figure 6:**
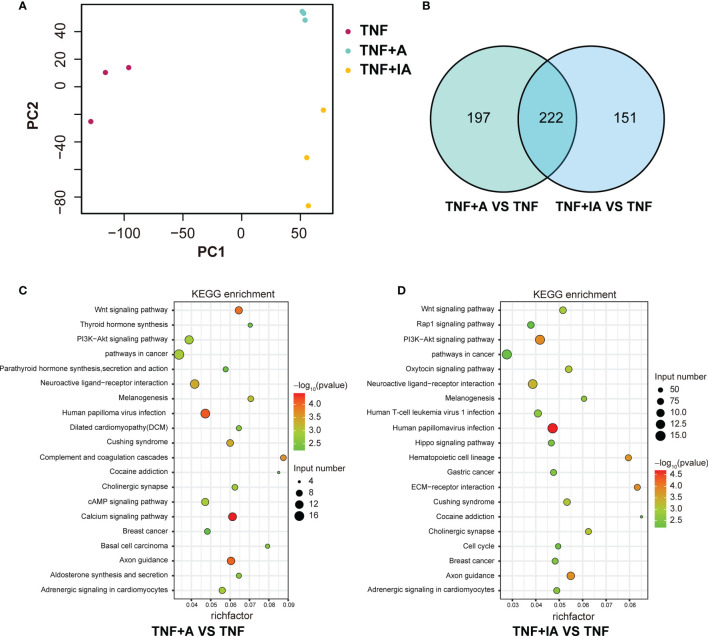
Comparison of gene expression abundance and KEGG enrichment in T, and TNF+A and TNF+IA groups. **(A)** The principal component analysis based on the RNA-Seq data. **(B)** Venn diagrams of differentially expressed transcripts. **(C)** The top 20 KEGG pathways enriched in TNF+A group compared to group TNF. **(D)** The top 20 KEGG pathways enriched in TNF+IA group compared to group TNF. TNF, cells challenged with 20 ng/mL Recombinant Susscrofa TNF-α for 48 hours. TNF+A, cells challenged with 20 ng/mL TNF-α for 40.5 h and then co-cultured with 10^8^ copies/mL active *A. muciniphila* for 7.5 h. TNF+IA, cells challenged with 20 ng/mL TNF-α for 40.5 h and then co-cultured with 10^9^ copies/mL inactive *A. muciniphila* for 7.5 h.

**Figure 7:**
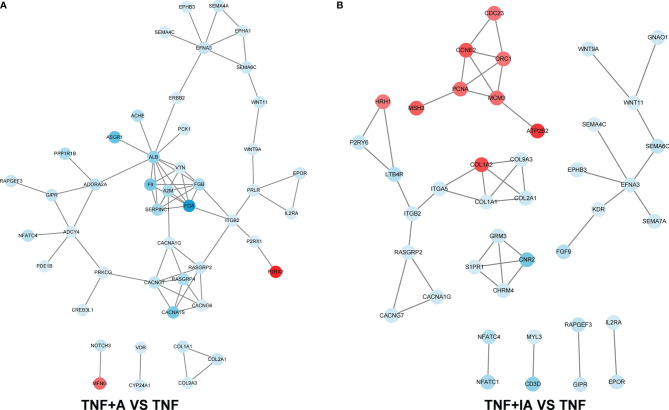
The protein-protein interaction network based on the KEGG annotation. **(A)** Change of protein abundance in TNF+A group compared to group TNF. **(B)** Change of protein abundance in TNF+IA group compared to group TNF. TNF, cells challenged with 20 ng/mL Recombinant Susscrofa TNF-α for 48 hours. TNF+A, cells challenged with 20 ng/mL TNF-α for 40.5 h and then co-cultured with 10^8^ copies/mL active *A. muciniphila* for 7.5 h. TNF+IA, cells challenged with 20 ng/mL TNF-α for 40.5 h and then co-cultured with 10^9^ copies/mL inactive *A. muciniphila* for 7.5 h.

As shown above, we found that both live and inactive *A. muciniphila* probably alleviated the inflammatory injury of IPEC-J2 induced by TNF-α, but the underlying pathway remains unclear. In order to find possible mechanism, a transcriptome analysis was performed. After removing low-quality reads, the average number of reads in each library was over 45 million. All filtered reads were aligned with the reference genome of Sus scrofa by Hisat2 software, and the transcripts identified in each sample (FPKM) were analyzed. The PCA targeting the top 20,000 genes with the highest expression level from the total 9 samples from the three groups revealed three corresponding independent clusters (**Figure 6A**). Further, a total of 197 DEGs were unique in TNF+A *vs* TNF, and 151 DEGs were only found in TNF+IA *vs* TNF (**Figure 6B**). The KEGG function of the filtered DEGs was then annotated using a KOBAS 3.0 (http://kobas.cbi.pku.edu.cn/kobas3/genelist/), and the first 20 paths with the lowest *p*-value were selected to generate bubble chart (**Figures 6C, D**). When comparing groups TNF+A and TNF, the different pathways involved in inflammatory response were identified as calcium signaling pathway and PI3K-Akt signaling pathway that also showed different between groups TNF+IA and TNF. The cell cycle pathway associated with cell proliferation and cycle also showed different between TNF+IA and TNF. According to the visualized protein interaction network (**Figure 7A, B**), the expression of proteins CACNA1S (Calcium channel, voltage-dependent, L type, alpha 1S subunit), CACNA1G (Calcium channel, voltage-dependent, T type, alpha 1G subunit), P2RX1 (Purinergic Receptor P2X 1) and P2RX2 (Purinergic Receptor P2X 2) in calcium signaling pathway, as well as the expression of proteins IL2RA (Interleukin-2 receptor subunit alpha), EPOR (Erythropoietin receptor) and PRLR (Prolactin Receptor) in PI3K-Akt signaling pathway were decreased (*P* < 0.05), and only the expression of ATP2B2 (ATPase plasma membrane calcium transporter) showed increased (*P* < 0.05) in TNF+A group compared to group TNF. Comparing with group TNF, the expression of proteins EPOR and IL2R (Interleukin-2 receptor) in PI3K-Akt signaling pathway and CDKN1C (Cyclin-dependent kinase inhibitor 1c) in cell cycle pathway showed decreased (*P* < 0.05), while the expression of proteins PCNA (Proliferating Cell Nuclear Antigen), CCNE2 (Cyclin E2), CDC23 (Cell division cycle 23), ORC1 (Origin Recognition Complex 1) and MCM3 (Mini-chromosome maintenance 3) in cell cycle pathway showed raised (*P* < 0.05).

## Discussion

Besides human beings, *A. muciniphila* is also found in the gut of other mammals such as guinea pigs, swines and rabbits (31–33). The effects of *A. muciniphila* on obesity and metabolic disorders have been widely discussed. However, its effect on the intestinal inflammation is controversial. In the gut of patients with inflammatory bowel disease (IBD) (34) or those with Crohn’s disease below 16 years of age (35), the abundant of *A. muciniphila* is reported to be reduced, indicating a positive role of this bacterium on gut health. But in two other studies, commensal *A. muciniphila* is observed to exacerbate the gut inflammation in *Salmonella typhimurium*-infected gnotobiotic mice (36) and was sufficient for promoting intestinal inflammation in germ-free *Il10^-/-^* mice (37). Another particularity of this bacterium is that some of its affects have been attributed to cellular components, such as proteins, which led us to think that similar effect could also be observed with inactivated cells. In the present study, we investigated the effect of *A. muciniphila* as alive or inactivated cultures on the IPEC-J2 cells with inflammation induced by Recombinant Susscrofa TNF-α to explore whether *A. muciniphila* benefit the health of IECs. To the best of our knowledge, the current study is the first to evaluate the role of both active and inactive *A. muciniphila* in an inflammatory model of IPEC-J2 cells.

Inflammatory response of IECs generally results in the occurrence of proinflammatory cytokines such as IL-1β, IL-6 and TNF-α (38, 39). The level of these cytokines is also widely used in clinical diagnosis as typical biomarkers of inflammation (40). In the current study, the mRNA level of *TNF-α* and *IL-1β* was remarkably increased in those cells treated with 20 ng/mL of Recombinant Susscrofa TNF-α for 48 hours, accompanied by visible lesion of cells under microscope. These results show that an inflammatory model of IPEC-J2 cells had been successfully induced by the recombinant TNF-α.

The potential to prevent or cure a certain disease is the main characteristic of NGPs different from FGPs, which makes most of the NGPs be classified to live biotherapeutics products (LBPs) (41). *A. muciniphila* is regarded as one of the most promising LBPs against metabolic diseases like obesity and diabetes. On the other hand, *A. muciniphila* may also play a role in the maintenance of intestinal homeostasis and improvement of intestinal inflammation. In patients with IBD, the relative abundance of *A. muciniphila* in the intestinal mucosa is decreased (42). Oral gavage of *A. muciniphila* is increases the level of 2-arachidonoylglycerol and in turn reduces the inflammatory responses in mice receiving a high-fat diet (10) and relieve dextran sulfate sodium (DSS)-induced colitis *via* its extracellular vesicles (43). In the current study, we not only showed the prevention of *A. muciniphila* as a LBP on the inflammation of IECs, but also showed that inactive *A. muciniphila* cells could be an available candidate against the inflammation of IECs. Whether firstly treated with 20 ng/mL of TNF-α for 40.5 h and followed by a treatment of active or inactive *A. muciniphila* for 7.5 h the mRNA level of proinflammatory cytokines was remarkably reduced along with the increased mRNA level of TJs. The epithelial permeability largely depends on adherin junctions and TJs. An increased intestinal permeability always contributes to the severity of some diseases, such as IBD and irritable bowel syndrome (44). The expression of genes encoding TJs is usually decreased when the function of IECs is impaired in case of inflammation (45–47). As a negative correlation has been found between TJs and the permeability of IPEC-J2 cells treated with TNF-α (45), the relative expression of TJs genes can be regarded as a marker for the damage of IECs. Our results thus showed a probable repair of *A. muciniphila* to the inflammatory damage of IECs, which was confirmed by the following analysis of flow cytometry. Apoptosis is a classical program of cell death, regulated by many factors such as TNF-α, IL-1β and caspase-2 (48–52). TNF-α has previously been reported to increase the apoptosis of IECs and cause the injury of intestinal barrier, which give rise to the occurrence of IBD. It was well grounded to hypothesize that *A. muciniphila* protecting the IECs from TNF-α-induced inflammation might be associated with the suppression of apoptosis. According to our results, the supplement of both active and inactive *A. muciniphila* after the treatment of TNF-α indeed reduced the early and total apoptosis of the IPEC-J2 cells. Similar results were also found in mice that the intestinal colonization of *A. muciniphila* strongly altered the expression of genes involved in the death of IECs at transcriptional level (16) and decreased the apoptosis of hepatocytes (53).

Limited to the descriptive results of the current study, the underlying mechanism of the positive effect of inactive *A. muciniphila* on the inflammatory IECs cannot be explained in detail. It has been shown that some cellular components, such as AmEVs, could increase the expression of TJs in Caco-2 cells and ultimately improve the integrity of intestinal barrier in mice with high-fat diet-induced diabetes (17). But this hypothesis cannot explain our results, as we used only cells but not total culture (and thus not extracellular metabolites) which were then autoclaved. The effects of *A. muciniphila* we observed is therefore might be due to a cellular component which is resistant to autoclave treatment. Although the profiles of scanning electron microscope showed that the integrity of inactive *A. muciniphila* cells were partially destroyed, some components of cell wall or membrane might still be biologically active, for instance, autoclave may not affect the structure of polysaccharides. Similarly, the specific membrane protein of *A. muciniphila* (Amuc_1100) has previously been reported to improve the intestinal barrier and can blunt colitis in mice (54, 55) even when the protein is pasteurized. Another clinical study shows that pasteurized 10^10^ of *A. muciniphila* is safe for human and can reduce the level of multiple inflammatory markers in serum (56), which proves the positive effect of inactive *A. muciniphila* on the systemic inflammation.

We further used transcriptome analysis to scan the potential signaling pathway of *A. muciniphila* in alleviating the TNF-α induced inflammation of IECs. We found that the treatment of active *A. muciniphila* down-regulated the expression of some key genes in calcium signaling pathway, such as CACNA1S, P2RX1 and P2RX2. It has been reported that under oxidative stress and other conditions, the increase of intracellular Ca^2+^ concentration can induce cell apoptosis and autophagy (57–59), while its decrease is associated with the remission of inflammation (60). Therefore, our results suggest that the apoptosis of TNF-α-challenged IECs may be inhibited by active *A. muciniphila* by reducing the influx of Ca^2+^ and increasing the outflow of Ca^2+^. In addition, although the treatment of inactive *A. muciniphila* was also found to reduce apoptosis of TNF-α-challenged IECs, the underlying mechanism may be different from that of active bacterium. We found that the expression of key genes involved in cell cycle, such as PCNA, CCNE2, CDC23, ORC1 and MCM3 in TNF-α-challenged IECs was up-regulated by the treatment of inactive *A. muciniphila*. All these genes play an important role in maintaining the growth and survival, the stability of genome and the life span of cells (61–70). At the same time, the expression of CDKN1C was down-regulated by inactive *A. muciniphila*, which may normalize the cell cycle (71–73). Another noteworthy path was PI3K-Akt signaling pathway. Studies have shown that PI3K-Akt signaling pathway is involved in the occurrence of ulcerative colitis and can be activated by intestinal inflammation (74, 75), and its inhibition can down-regulate the expression of nuclear transcription factors-κB (NF-κB) to reduce the expression of proinflammatory cytokines (76, 77). We found that the treatment of both active and inactive *A. muciniphila* down-regulated the expression of upstream receptors (IL2RA, EPOR, PRLR and/or PIK3R5) in PI3K-Akt signaling pathway, which may directly decline the expression of cytokines like TNF- α, IL-8, IL-1β and IL-6 in these TNF-α-challenged cells.

## Conclusions

Our study suggests that active and autoclaved inactive *A. muciniphila* improved the TNF-α-induced inflammation of IECs by reducing the expression of pro-inflammatory cytokines and increasing the expression of TJs, which might be associated with the decreased apoptosis. Although both active and inactive *A. muciniphila* showed significant remission of inflammation and apoptosis of TNF-α-challenged IECs, the underlying mechanism may be different. Active *A. muciniphila* may decline cell apoptosis by down-regulating the expression of key genes in calcium signaling pathway, while inactive *A. muciniphila* may reduce cell apoptosis by up-regulating the expression of key genes in cell cycle signaling pathway. Both live and inactive *A. muciniphila* may alleviate the inflammation of IECs by down-regulating the expression of PI3K upstream receptor genes. The specific effect of *A. muciniphila* on inflammatory IECs indicates that it may be a promising candidate of NGP for piglets or other animals with similar physiology and anatomy to cure inflammatory. However, further *in vivo* researches are still needed to explore the underlying mechanism of active and inactive *A. muciniphila* on the inflammation of IECs.

## Data Availability Statement

The datasets presented in this study can be found in online repositories. The names of the repository/repositories and accession number(s) can be found below: https://www.ncbi.nlm.nih.gov/genbank/, PRJNA752872.

## Author Contributions

YL designed the experiment and wrote the manuscript. CL and KX finished the experiment and the real-time PCR analysis. HL conducted transcriptome analysis and helped finish the experiment. ED and JH helped design the experiment. LL provided the strain and helped design the experiment. JC, GT, AW, ZH, and DC helped finish the laboratory analysis. BY, ZH, PZ, XM, JY, and JL helped revise the manuscript. HY, QW, and HW helped collect the references and purchase reagents. JT helped analyze real-time PCR data. All authors contributed to the article and approved the submitted version.

## Funding

This work was supported by Adisseo and the National Natural Science Foundation of China (NSFC, grant number 31872369 and 32072743).

## Conflict of Interest

The authors declare that the research was conducted in the absence of any commercial or financial relationships that could be construed as a potential conflict of interest.

## Publisher’s Note

All claims expressed in this article are solely those of the authors and do not necessarily represent those of their affiliated organizations, or those of the publisher, the editors and the reviewers. Any product that may be evaluated in this article, or claim that may be made by its manufacturer, is not guaranteed or endorsed by the publisher.
